# Clinical performance of Roche cobas 6800, Luminex ARIES, MiRXES Fortitude Kit 2.1, Altona RealStar, and Applied Biosystems TaqPath for SARS‐CoV‐2 detection in nasopharyngeal swabs

**DOI:** 10.1002/jmv.26940

**Published:** 2021-03-30

**Authors:** Chun Kiat Lee, Jason Wei Ming Tham, Siyu Png, Chean Nee Chai, Shu Chi Ng, Eunice Jia Min Tan, Li Jie Ng, Rui Ping Chua, Musa Sani, Yiqi Seow, Gabriel Yan, Julian Tang

**Affiliations:** ^1^ Department of Laboratory Medicine National University Health System Singapore Singapore; ^2^ Institute of Bioengineering and Nanotechnology Agency for Science, Technology and Research Singapore Singapore; ^3^ Department of Medicine National University Health System Singapore Singapore; ^4^ Department of Respiratory Sciences University of Leicester Leicester UK

**Keywords:** coronavirus, COVID‐19, molecular diagnostics, pandemic, SARS‐CoV‐2

## Abstract

We compared the performance of five assays for severe acute respiratory syndrome coronavirus 2 (SARS‐CoV‐2) detection on nasopharyngeal swab samples: Roche “cobas,” Luminex “ARIES,” MiRXES “Fortitude,” Altona “RealStar,” and Thermo Fisher Scientific “TaqPath.” A total of 94 nasopharyngeal swab samples were obtained from 80 confirmed coronavirus disease 2019 cases in the first 2 weeks of illness (median, 7 days; range, 2–14 days) and 14 healthy controls. After collection, all samples were transported to the hospital clinical laboratory within 24 h. These samples were tested on all five assays within 3 days of sample receipt. Of the 94 samples, 69 yielded the same result on all platforms, resulting in an agreement of 73.4% (69 of 94). Of these, 14 were the healthy control swabs which all tested negative, demonstrating good specificity across all platforms. The ARIES assay had the lowest detection rate (68.8%), followed by Fortitude (85.0%), RealStar (86.3%), cobas (95.0%), and TaqPath (100%). Statistically significant differences were observed for ARIES, Fortitude, and RealStar when compared against the best performing TaqPath using McNemar's *χ* ^2^ test. A consensus result was established based on the results obtained by the cobas, Fortitude, RealStar, and TaqPath. Six discrepancies had failed to reach a consensus and were adjudicated using the Cepheid Xpert Xpress SARS‐CoV‐2. Overall, the TaqPath and cobas assays were the most sensitive at detecting their designated SARS‐CoV‐2 gene targets. On the other hand, the ARIES assay was the least sensitive, thus warranting the need for assay re‐optimization before go‐live at the testing laboratory.

## 1 INTRODUCTION

The severe acute respiratory syndrome coronavirus 2 (SARS‐CoV‐2, Family *Coronaviridae*, genus *Betacoronavirus*, species *Severe acute respiratory syndrome–related coronavirus*) is the causative agent for coronavirus disease 2019 (COVID‐19). Since the beginning of the COVID‐19 pandemic in January 2020,^1^ multiple commercial molecular diagnostic assays have become available for its diagnosis.^2^ Here, we compare the performance of five kits for SARS‐CoV‐2 RNA detection (Table 1), namely: the Roche cobas SARS‐CoV‐2 (herein referred as “cobas”; Roche Molecular Systems), the Luminex ARIES SARS‐CoV‐2 (herein referred as “ARIES”; Luminex Corp.), the MiRXES Fortitude Kit 2.1 (herein referred as “Fortitude“; MiRXES Pte. Ltd.), the Altona RealStar SARS‐CoV‐2 1.0 (herein referred as “RealStar”; Altona diagnostics GmbH), and the Applied Biosystems TaqPath COVID‐19 Combo Kit (herein referred as “TaqPath”; Thermo Fisher Scientific) on nasopharyngeal swabs (NPS) collected in universal transport medium (UTM, Copan Diagnostics Inc.). The cobas, ARIES, RealStar, and TaqPath assays have received Emergency Use Authorisation (EUA) status in the United States while the Fortitude has received Provisional Authorisation in Singapore.^2^

**Table 1:** Characteristics of the five assays evaluated in this study

Assay (manufacturer)	Cobas SARS‐CoV‐2 (Roche “cobas”)	ARIES SARS‐CoV‐2 (Luminex “ARIES”)	Fortitude Kit 2.1 (MiRXES “Fortitude“)	RealStar SARS‐CoV‐2 1.0 (altona “RealStar“)	TaqPath COVID‐19 Combo Kit (Thermo Fisher Scientific “TaqPath”)
Target gene/s	ORF1ab (SARS‐CoV‐2)	ORF1ab (SARS‐CoV‐2)	ORF1ab region 1 (SARS‐CoV‐2)	S (SARS‐CoV‐2)	ORF1ab (SARS‐CoV‐2)
	E (pan‐sarbecovirus)	N (SARS‐CoV‐2)	ORF1ab region 2 (SARS‐CoV‐2)	E (pan‐sarbecovirus)	N (SARS‐CoV‐2)
					S (SARS‐CoV‐2)
EUA status	Yes	Yes	No	Yes	Yes
No. of samples tested	94	94	94	94	94
Positive concordance with the TaqPath COVID‐19 Combo Kit (%)	76/80 (95%)	55/80 (69%)	68/80 (85%)	69/80 (86%)	NA
Positive concordance with the consensus results (%)	74/74 (100%)	55/74 (74%)	68/74 (92%)	69/74 (93%)	74/74 (100%)
Negative concordance with the TaqPath COVID‐19 Combo Kit (%)	14/14 (100%)	14/14 (100%)	14/14 (100%)	14/14 (100%)	NA
Negative concordance with the consensus results (%)	18/20 (90%)	20/20 (100%)	20/20 (100%)	20/20 (100%)	14/20 (63%)
PPA/NPA/Cohen's *κ* coefficient with the TaqPath COVID‐19 Combo Kit (95% CI)	PPA: 95% (88%–99%)	PPA: 69% (57%–79%)	PPA: 85% (75%–92%)	PPA: 86% (77%–93%)	NA
	NPA: 100% (77%–100%)	NPA: 100% (77%–100%)	NPA: 100% (77%–100%)	NPA: 100% (77%–100%)	
	*κ*: 0.85 (0.71–0.99)	*κ*: 0.40 (0.23–0.56)	*κ*: 0.63 (0.45–0.81)	*κ*: 0.65 (0.47–0.83)	
PPA/NPA/Cohen's *κ* coefficient with the consensus results (95% CI)	PPA: 100% (95%–100%)	PPA: 74% (63%–84%)	PPA: 92% (83%–97%)	PPA: 93% (85%–98%)	PPA: 100% (95%–100%)
	NPA: 90% (68%–99%)	NPA: 100% (83%–100%)	NPA: 100% (83%–100%)	NPA: 100% (83%–100%)	NPA: 70% (46%–88%)
	κ: 0.93 (0.84–1.00)	κ: 0.55 (0.39–0.71)	*κ*: 0.82 (0.70–0.96)	*κ*: 0.85 (0.73–0.98)	*κ*: 0.78 (0.62–0.94)

Abbreviations: COVID‐19, coronavirus disease 2019; NPA, negative percent agreement; PPA, positive percent agreement; SARS‐CoV‐2, severe acute respiratory syndrome coronavirus 2.

## 2 METHODS

A total of 94 NPS samples were obtained from 80 confirmed COVID‐19 cases in the first 2 weeks of illness (median, 7 days; range, 2–14 days) and 14 healthy controls. After collection, all NPS were transported to the hospital clinical laboratory within 24 h.

Ethics approval was granted by the National Healthcare Group Domain‐specific review board (NHG ROAM ref. 2020/00337). Statistical analyses were performed using the R version 3.6.0 (with *p* < .05 being statistically significant).

Of the five platforms selected for this comparison study, only the cobas and the ARIES are sample‐to‐result platforms providing a fully automated and complete walk‐away solution. All testing for the cobas and the ARIES was performed according to the manufacturers' instructions.

The cobas targets the open reading frame 1ab (ORF1ab) nonstructural region for specific SARS‐CoV‐2 detection and a conserved region of the structural protein envelope (E) gene for pan‐sarbecovirus detection while the ARIES targets both the ORF1ab and the nucleoprotein (N) genes for specific SARS‐CoV‐2 detection (Figure S1).

For the remaining assays, total nucleic acid was extracted from 400 µl of UTM on the KingFisher Flex instrument (Thermo Fisher Scientific) and eluted in 90 µl of elution buffer. All Fortitude and RealStar reverse‐transcription polymerase chain reactions (RT‐PCRs) were run on the LightCycler 480 Instrument II (Roche Molecular Systems) while all TaqPath RT‐PCRs were run on the Applied Biosystems 7500 Fast Real‐Time PCR Instrument (Thermo Fisher Scientific).

The Fortitude amplifies and detects two different regions within the ORF1ab gene for specific SARS‐CoV‐2 detection while the RealStar targets the spike protein (S) gene for specific SARS‐CoV‐2 detection and the E gene for pan‐sarbecovirus detection. The TaqPath employs three different gene targets (ORF1ab, N, and S genes) for specific SARS‐CoV‐2 detection (Figure S1).

To ensure a fair comparison, all tests (inclusive of discrepancy resolution) were completed within 3 days on fresh clinical NPS samples stored at 4°C, to ensure equivalent sample quality upon testing. On Day 3, discrepancy resolution was conducted using the Cepheid Xpert Xpress SARS‐CoV‐2 (“Xpert”, Cepheid) which targets the N2 region of the N gene for specific SARS‐CoV‐2 detection and a conserved region of the E gene for pan‐sarbecovirus detection.

## 3 RESULTS

Following testing of 94 NPS samples, 69 yielded the same result on all platforms, resulting in an agreement of 73.4% (69 of 94). Of these, 14 were the healthy control swabs which all tested negative, demonstrating good specificity across all platforms. In contrast, only 68.8% (55 of 80) of the swabs from the confirmed COVID‐19 cases tested positive across all five platforms.

Notably, the ARIES assay had the lowest detection rate (68.8%; 55 of 80). This was followed by Fortitude (85.0%, 68 of 80), RealStar (86.3%, 69 of 80), cobas (95.0%, 76 of 80), and TaqPath (100%, 80 of 80) (Figure S2). McNemar's *χ* ^2^ test was used to compare the performance of the all assays against the best performing TaqPath assay. Significant differences were observed for ARIES (McNemar's *χ* ^2^ = 23.04, *df* = 1, *p* value =1.59e − 06), Fortitude (10.08, 1, 0.0015), and RealStar (9.09, 1, 0.0026). No statistically significant difference was observed for cobas (2.25, 1, 0.13). Using TaqPath as reference (Table 1), the cobas assay had a *κ* coefficient of 0.85 (95% CI, 0.71–0.99; strong agreement), positive percent agreement (PPA) of 95% (95% CI, 88%–99%), and negative percent agreement (NPA) of 100% (95% CI, 77%–100%). Similarly, for the ARIES: *κ* = 0.40 (95% CI, 0.23–0.56, weak agreement); PPA = 69% (95% CI, 57%–79%), NPA = 100% (95% CI, 77%–100%); Fortitude: *κ* = 0.63 (95% CI, 0.45–0.81, moderate agreement), PPA = 85% (95% CI, 75%–92%), NPA = 100% (95% CI, 77%–100%); RealStar assay, the *κ* coefficient was 0.65 (95% CI, 0.47–0.83; moderate agreement), PPA was 86% (95% CI, 77%–93%), and NPA was 100% (95% CI, 77%–100%).

We then performed more complex concordance analyses involving the establishment of a consensus result which was defined as the result obtained by at least three of the four assays. The four assays comprised the cobas, Fortitude, RealStar, and TaqPath. The ARIES was excluded as it had shown poor agreement when compared against the others. A total of six discrepancies had failed to reach a consensus but were subsequently adjudicated using the Xpert assay. Of the six discrepancies, five were reclassified as positives (detected: cobas, TaqPath, and Xpert; not detected: Fortitude and RealStar) and one was reclassified as negative (detected: cobas and TaqPath; not detected: Fortitude, RealStar, and Xpert). Other cross‐comparison results are shown in Table 1.

Table 2 provides an overview of the cycle threshold (CT) values for the 25 discordant samples across all gene targets. Except for ARIES, there was complete concordance across all assays for 13 samples with a median TaqPath N gene CT value of 33.45 (interquartile range [IQR], 31.56–35.17). The remaining discordant samples had a median TaqPath N gene CT value of 36.67 (IQR, 34.79–37.24), suggesting a low SARS‐CoV‐2 viral burden which could be near the detection limit of the different gene targets. The TaqPath N gene was found to be the most sensitive among the various gene targets for low viral load samples, followed by cobas E gene and RealStar S gene (TaqPath N > cobas E > RealStar S genes). A notable difference in sensitivity was also observed between the TaqPath gene targets. Among the 25 samples missed by ARIES, 15 (60%; 15 of 25) were positive for the TaqPath N gene but negative for the TaqPath ORF1ab and S genes (Table 2).

**Table 2:** An overview of the cycle threshold (CT) values for the 25 discordant results between the five assays evaluated in this study

Assay (manufacturer)	cobas SARS‐CoV‐2 (Roche “cobas”)		ARIES SARS‐CoV‐2 (Luminex “ARIES”)		Fortitude Kit 2.1 (MiRXES “Fortitude”)		RealStar SARS‐CoV‐2 1.0 (altona “RealStar”)		TaqPath COVID‐19 Combo Kit (Thermo Fisher Scientific “TaqPath”)		
Target gene/s	ORF1ab	E	ORF1ab	N	ORF1ab region 1	ORF1ab region 2	S	E	ORF1ab	N	S
Cycle threshold	‐	37.39	‐	‐	‐	‐	‐	‐	‐	37.61	‐
	‐	‐	‐	‐	‐	‐	‐	‐	‐	36.11	‐
	‐	36.88	‐	‐	‐	‐	‐	‐	‐	37.43	‐
	‐	36.54	‐	‐	‐	‐	‐	‐	‐	34.72	‐
	33.50	35.49	‐	‐	32.21	32.97	30.66	29.92	32.64	29.67	32.37
	31.05	32.81	‐	‐	32.27	33.25	30.02	29.49	34.11	31.15	34.44
	‐	36.38	‐	‐	33.55	34.44	30.84	30.19	36.95	31.50	‐
	32.67	34.36	‐	‐	34.06	35.06	32.22	31.58	35.85	32.96	37.38
	‐	38.78	‐	‐	‐	‐	‐	‐	‐	37.17	‐
	31.40	32.47	‐	‐	32.87	33.44	31.79	31.19	35.87	31.56	34.53
	33.09	34.41	‐	‐	34.32	35.18	32.55	31.88	34.91	33.45	36.04
	34.26	37.07	‐	‐	‐	36.12	34.17	‐	‐	35.17	‐
	32.46	37.59	‐	‐	‐	‐	‐	‐	‐	36.78	‐
	32.51	34.38	‐	‐	34.60	35.06	32.73	32.01	35.97	32.20	39.04
	33.66	36.78	‐	‐	35.00	36.27	33.70	32.86	‐	36.16	‐
	32.25	35.29	‐	‐	‐	‐	‐	‐	35.49	33.09	37.74
	‐	36.50	‐	‐	32.80	33.80	30.78	30.22	38.97	33.94	38.62
	‐	‐	‐	‐	‐	‐	‐	‐	‐	36.83	‐
	‐	‐	‐	‐	‐	‐	‐	‐	‐	38.71	‐
	35.12	‐	‐	‐	35.01	36.07	34.95	33.99	‐	35.35	‐
	‐	38.03	‐	‐	35.15	‐	35.46	34.69	‐	37.61	‐
	‐	37.91	‐	‐	‐	‐	‐	‐	‐	36.56	‐
	‐	‐	‐	‐	‐	‐	‐	‐	‐	36.98	‐
	32.84	34.48	‐	‐	33.67	34.61	32.93	32.23	36.14	33.81	37.82
	33.02	36.33	‐	‐	‐	‐	32.76	32.08	‐	34.81	‐
% positive detection	13/25	**20/25**	0/25	0/25	12/25	12/25	**14/25**	13/25	10/25	**25/25**	9/25
	(52%)	**(80%)**	(0%)	(0%)	(48%)	(48%)	**(56%)**	(52%)	(40%)	**(100%)**	(36%)

Overall, the TaqPath and cobas assays were the most sensitive at detecting their designated SARS‐CoV‐2 gene targets (Table 1). On the other hand, the ARIES assay was the least sensitive, warranting the need for further assay re‐optimization before go‐live at the testing laboratory.

## 4 DISCUSSION

SARS‐COV‐2 subgenomic RNAs are required for efficient viral protein production and these are generated through discontinuous RNA synthesis, linking the 5' leader sequence with the appropriate open reading frames.^3^ As such, in cells with replicating SARS‐COV‐2, the abundance of copies will trend towards the 3' end of the genome. In terms of relative abundance within the replicating cells, there will be most copies of the N gene RNA, followed by E, S, and ORF1ab gene RNAs. The abundance of the N gene target was also noted earlier with SARS‐CoV, though the enhanced assay sensitivity based on this target was only transient and decreased during the course of clinical disease.^4^

In the current study, Figure S3 compares the CT of the different gene targets with an increasing number of days post‐symptom onset. Generally, the testing trend is consistent across all five assays, showing increasing CT values which may reflect host immune clearance of the SARS‐CoV‐2 over time, that is, decreasing viral load.

Thus in circumstances where very low numbers or no virions are being produced (i.e. in the early onset of infection or at the tail‐end of a resolving infection), the more sensitive tests that target the N gene may still show some viral RNA compared to other tests detecting other less sensitive targets (i.e. N > E > S>ORF1ab genes), assuming equal amplification efficiencies.

With the exception of the ARIES, which may suffer from other issues given the low number of positives detected by either the N or ORF1ab gene targets, this trend can be seen in our results, with TaqPath N gene target detecting 80 of 80 compared to RealStar and cobas E gene targets, detecting 69 of 80 and 75 of 80, respectively, followed by the assays using the S and ORF1ab gene targets (see Figure S2). Within each assay, similar trends were also seen with TaqPath N gene target being more sensitive than TaqPath S gene target and the cobas E gene target being more sensitive than the ORF1ab gene target. Even in the problematic ARIES kit, this trend was also observed.

Although numerous rapid tests have now been produced since the beginning of the COVID‐19 pandemic,^5^, ^6^ “gold standard” laboratory‐based assays are still required to reliably confirm the presence or absence of SARS‐CoV‐2 infection in patients, particularly now with the emergence of reinfection cases defined both clinically and in the laboratory,^7^, ^8^, ^9^ to guide and optimize public health interventions to control the ongoing spread of COVID‐19.

## CONFLICT OF INTERESTS

The authors declare that htere are no conflict of interest.

## AUTHOR CONTRIBUTIONS

Chun Kiat Lee: *Conceptualization, formal analysis, writing—Original Draft, Supervision*. Jason Wei Ming Tham: *Investigation, project administration*. Siyu Png: *Investigation, project administration*. Chean Nee Chai: *Investigation, project administration*. Shu Chi Ng: *Investigation, project administration*. Eunice Jia Min Tan: *Investigation, project administration*. Li Jie Ng: *Investigation*. Rui Ping Chua: *Investigation*. Musa Sani: *Investigation*. Yiqi Seow: *Writing—original draft, writing—review & editing*. Gabriel Yan: *Writing—review & editing. Julian W Tang: Validation, writing—original draft, review & editing, supervision*.

### PEER REVIEW

The peer review history for this article is available at https://publons.com/publon/10.1002/jmv.26940

## Supporting information

Supporting information.

Supporting information.

Supporting information.

## Data Availability

The data that support the findings of this study are available from the corresponding author upon reasonable request.
