# Supplementary figures and images for: Clinical performance of Roche cobas 6800, Luminex ARIES, MiRXES Fortitude Kit 2.1, Altona RealStar, and Applied Biosystems TaqPath for SARS‐CoV‐2 detection in nasopharyngeal swabs
Source: J Med Virol. 2021 Mar 30;93(7):4603–7. doi: 10.1002/jmv.26940 (PMC8250924; doi:10.1002/jmv.26940)

**Figure S1.** The SARS-CoV-2 genome showing the location of the various assay target genes.


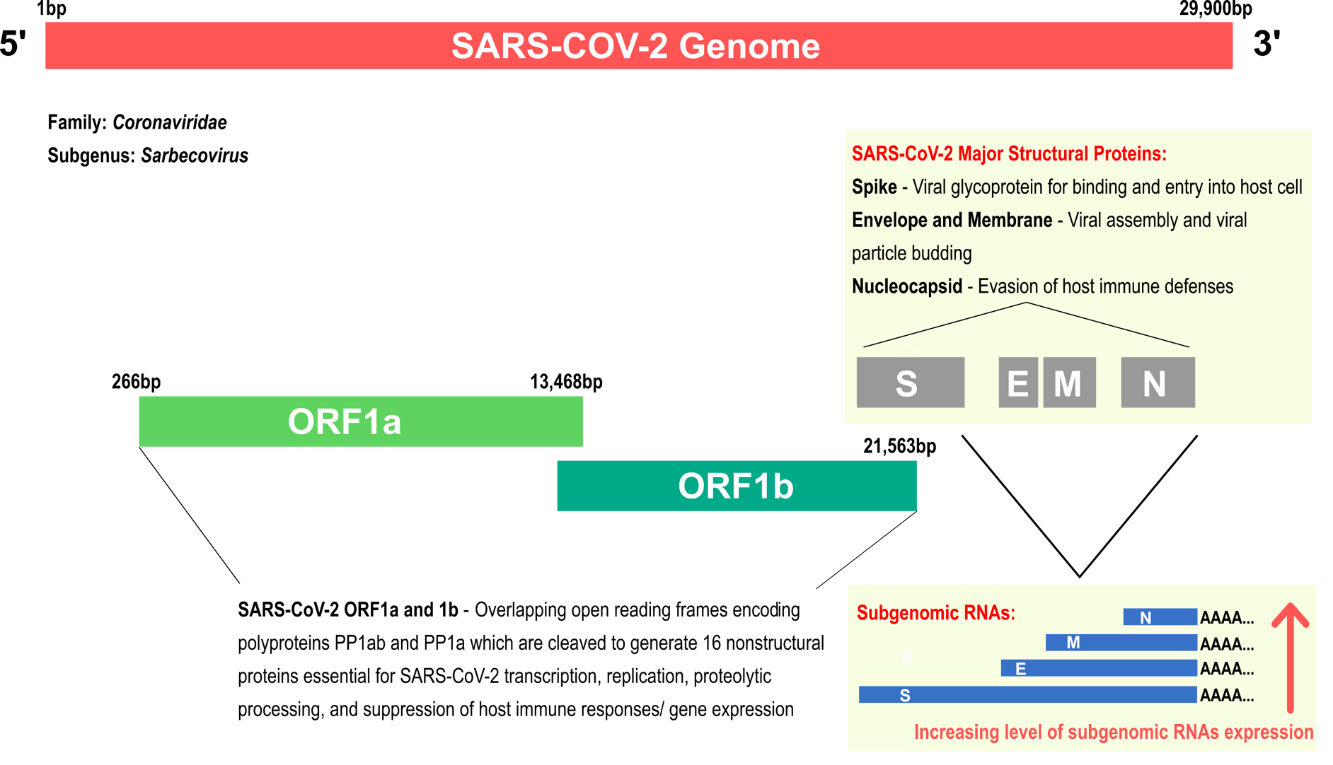

Supplement: Supplementary file 1 — Supporting information. [file JMV-93-4603-s002.doc]
