# Supplementary material for: Clinical performance of Roche cobas 6800, Luminex ARIES, MiRXES Fortitude Kit 2.1, Altona RealStar, and Applied Biosystems TaqPath for SARS‐CoV‐2 detection in nasopharyngeal swabs
Source: J Med Virol. 2021 Mar 30;93(7):4603–7. doi: 10.1002/jmv.26940 (PMC8250924; doi:10.1002/jmv.26940)

**Figure S2.** Comparative performances of the 80 known SARS-CoV-2 positive swabs. The box-whisker plots below show the median (horizontal line in the boxes) CT values, with the first quartile (Q1, 25^th^ percentile) and third quartile (Q3, 75^th^ percentile) CT values represented by the lower and upper dimensions of the box, together with the minimum and maximum CT values (bottom and top line extensions). Overall, the TaqPath and cobas assays were the most sensitive at detecting their designated SARS-CoV-2 gene targets. The plots below were drawn using R version 3.6.0.


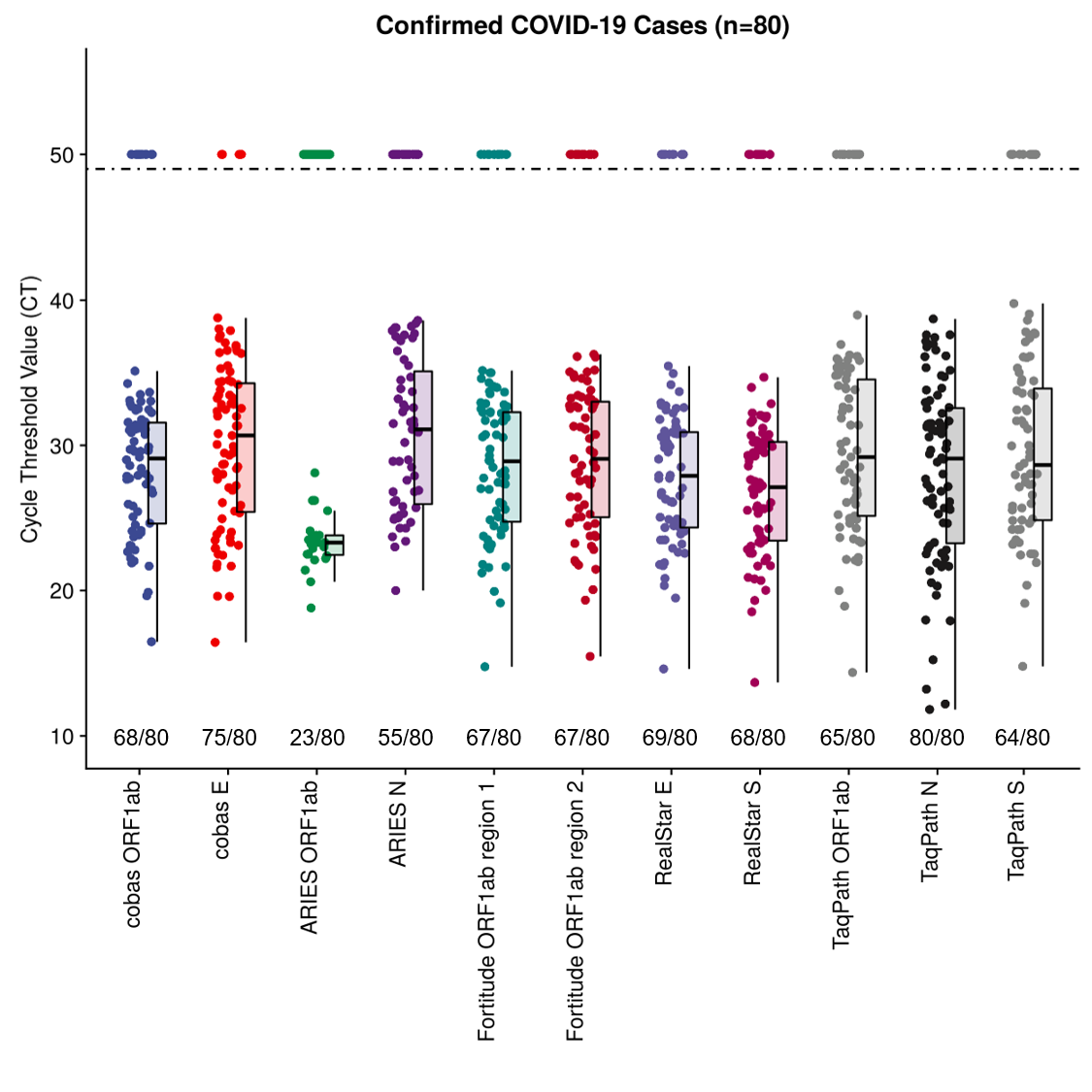

Supplement: Supplementary file 2 — Supporting information. [file JMV-93-4603-s001.doc]
