# Supplementary material for: Clinical performance of Roche cobas 6800, Luminex ARIES, MiRXES Fortitude Kit 2.1, Altona RealStar, and Applied Biosystems TaqPath for SARS‐CoV‐2 detection in nasopharyngeal swabs
Source: J Med Virol. 2021 Mar 30;93(7):4603–7. doi: 10.1002/jmv.26940 (PMC8250924; doi:10.1002/jmv.26940)
Supplement: Supplementary file 3 — Supporting information. [file JMV-93-4603-s003.doc]

**Figure S3.** Comparative performance of the five assays with increasing number of days post-symptom onset – by individual gene target (standard error of the regression is represented by the shaded area). The plots below were drawn using R version 3.6.0.

**
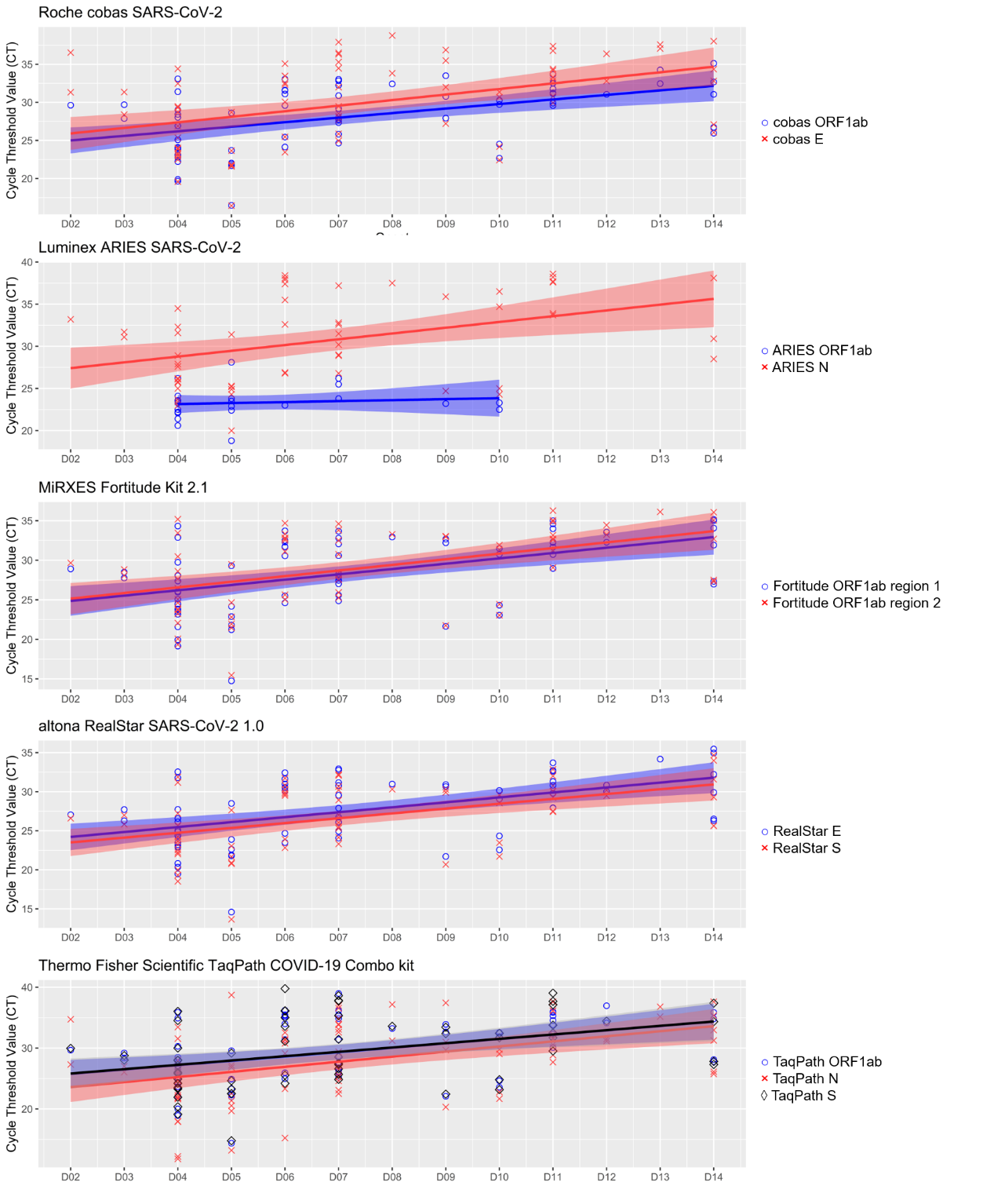
**
